# Piperine inhibits biofilm formation and efflux activity and dysregulates lipid metabolism in *Mycobacterium abscessus*

**DOI:** 10.1371/journal.pone.0341420

**Published:** 2026-01-22

**Authors:** Toe Ko Ko Htay, Matthew R. Hathaway, Jaiyanth Daniel

**Affiliations:** Department of Biological Sciences, Purdue University Fort Wayne, Fort Wayne, Indiana, United States of America; University of Nebraska Medical Center, UNITED STATES OF AMERICA

## Abstract

The nontuberculous *Mycobacterium abscessus* is a human pathogen that causes chronic lung infections and soft tissue infections. The bacterium forms biofilms and efflux pumps contribute to its tolerance of antibiotics. Efflux pumps also transport lipids and other molecules to the bacterial outer cell surface for biofilm formation. The effects of piperine, an alkaloid derived from black pepper, on biofilm formation, efflux activity and lipid biosynthesis in *M. abscessus* have not been reported. We report that, at sub-minimum inhibitory concentration levels, piperine inhibits biofilm formation in *M. abscessus* by more than 90%. We investigated lipid biosynthesis from exogenously supplied radiolabeled ^14^C-palmitic acid in *M. abscessus* during its log-phase growth and during biofilm formation and examined the effects of piperine. We report that piperine dysregulates the biosynthesis of major lipids in *M. abscessus* during biofilm formation. Piperine inhibited the biosynthesis of the neutral storage lipid triacylglycerol during biofilm formation by nearly 80% and the biosynthesis of the polar lipid trehalose monomycolate by 50%. In contrast, piperine stimulated the biosynthesis of the major polar lipid phosphatidylethanolamine during biofilm formation. Piperine inhibited efflux activity in *M. abscessus* by nearly 70%. Piperine enhanced the efficacies of four commonly used antibiotics used to treat *M. abscessus* infections. The minimum inhibitory concentration of clarithromycin was decreased by more than 16-fold by piperine and that of amikacin and cefoxitin by about 5-fold. The efficacy of ciprofloxacin was improved by more than 2-fold by piperine. This is the first report on the effects of piperine on lipid biosynthesis, efflux activity and biofilm formation in *M. abscessus* that highlights the potential importance of piperine as an adjunct therapy to treat nontuberculous mycobacterial infections.

## 1. Introduction

The non-tuberculous *Mycobacterium abscessus* is a human pathogen that causes increasing numbers of soft tissue and pulmonary infections [1,2]. This pathogen is also highly tolerant to antibiotics and is an emerging health threat [1]. Therefore, the development of adjunct therapies that increase the efficacies of currently used antibiotics is a critical need.

Microorganisms exist as biofilms in their natural state in the environment and humans and all organisms are colonized by biofilm-forming microbes [3]. Several mycobacterial species, including nontuberculous mycobacteria, have been shown to form biofilms which are comprised of lipids, polysaccharides, proteins and eDNA [4]. *M. abscessus* biofilms are primarily comprised of lipids such as free mycolic acids in the extracellular matrix [5–7]. Mycolic acids are very long-chain fatty acids in the mycobacterial cell envelope. Lipids form a major component of the extracellular matrix of the biofilms of *M. abscessus* [8]. In our previous study we showed that, in *M. abscessus* biofilms, the biosynthesis of polar membrane lipids such as phosphatidylethanolamine (PE) and CL from exogenous fatty acids was inhibited by the putative efflux pump inhibitor naphthylmethylpiperazine [9].

*M. abscessus* encodes efflux pumps that have been shown to be involved in antibiotic resistance [10]. The MmpL family of efflux pumps have been linked to resistance to antibiotics such as clofazimine and bedaquiline in *M. abscessus* [11]. Recently, it was shown that MAB_2303 was directly involved in the efflux of linezolid in *M. abscessus* [12]. Efflux pump inhibitors have been shown to act synergistically with antibiotics against *M. abscessus* [13]. Bacterial efflux pumps could also extrude a diverse array of molecules in nonclinical environments inhabited by the microorganisms [14]. Bacterial efflux pumps are postulated to play multiple roles in biofilm formation that includes the transport of extracellular polymeric substances that constitute the biofilm matrix [15]. Efflux pumps are involved in the formation of the mycobacterial cell envelope and in mycobacterial biofilm formation which are critical to the intrinsic drug tolerance of mycobacteria [16,17]. In *Mycobacterium tuberculosis*, several studies have examined the inhibition of efflux pumps belonging to the various superfamilies by efflux pump inhibitors [18]. Efflux pump inhibitors have been demonstrated to be effective as adjunct therapies that enhance the effectiveness of antibiotics against multidrug resistant *M. tuberculosis* and nontuberculous mycobacteria [19,20].

Piperine, an alkaloid molecule derived from black pepper (*Piper nigrum*) and long pepper (*Piper longum*), has been shown to have antimicrobial activities [21,22]. A systematic review of a large number of studies on piperine highlighted its antibacterial activities including its ability to inhibit biofilm formation and efflux activity [23]. Earlier studies have shown that piperine is an inhibitor of multidrug efflux pumps in *Mycobacterium tuberculosis* and *Mycobacterium smegmatis* [24,25]. Additionally, it has been demonstrated that piperine acts synergistically with anti-tuberculosis drugs [26]. Piperine also enhanced the efficacy of clarithromycin against *Mycobacterium avium* [27]. Furthermore, piperine was demonstrated to inhibit biofilm formation in *M. tuberculosis* [28]. Piperine is not toxic to mammalian cells and has been shown to improve the efficacy of rifampicin against *M. tuberculosis* in mice [29]. The new anti-tuberculosis drug risorine, which contained piperine in combination with rifampicin and isoniazid, was highly effective in treating drug-susceptible tuberculosis in human patients [30]. A recent study showed that piperine enhanced the efficacy of clarithromycin against rapidly growing mycobacteria including *M. abscessus* [31]. The efficacy of piperine in controlling *M. abscessus* infection has not been reported. Based on the reports above, we postulated that piperine might affect biofilm formation, efflux activity and lipid biosynthesis in *M. abscessus*. To our knowledge, there are no reports on the effects of piperine on lipid biosynthesis associated with biofilm formation in any bacteria.

Therefore, in this study, we investigated the effects of piperine on the growth, biofilm formation, efflux activity, lipid biosynthesis and antibiotic efficacy in *M. abscessus*. We report here that piperine was highly effective in inhibiting the growth in liquid culture, biofilm formation and efflux activity in *M. abscessus*. Furthermore, piperine enhanced the efficacies of four commonly used antibiotics used to treat nontuberculous mycobacterial infections. We also observed that piperine dysregulated lipid biosynthesis from exogenous fatty acid precursor during biofilm formation. This is the first report on the effects of piperine on biofilm formation, efflux activity and lipid biosynthesis during biofilm formation in *M. abscessus.*

## 2. Materials and methods

### 2.1. Mycobacterial conditions

*Mycobacterium abscessus (Mab)* ATCC 19977 cells were obtained from the ATCC, Manassas, VA, USA. Cells stored as glycerol stock at −80 °C were grown in Middlebrook 7H9 medium containing 0.05% (w/v) Tween 80, 10% (v/v) Middlebrook albumin dextrose catalase (ADC) enrichment with shaking at 37 °C to mid-log phase when optical density at 600 nm (OD_600_) was between 0.6–0.8. *M. abscessus* was induced to form biofilms by diluting the log-phase culture 1:100 into our biofilm-stimulating medium lacking Tween 80 but supplemented with 2% (w/v) ADC and 0.5% (w/v) glucose and incubation without shaking, as described previously [9].

### 2.2. Chemicals and reagents

Difco Middlebrook 7H9 broth (Becton, Dickinson and Company, Sparks, MD) and all other chemicals used were of analytical grade. Mueller-Hinton broth (Oxoid/ Thermo Fisher Scientific, UK) was supplemented with salts to provide 20–25 mg/L calcium and 10–12 mg/L magnesium to make cation-adjusted Mueller-Hinton broth (CAMHB). The following antimicrobials were used in this study: Piperine (PIP; 98%, Cat. No. A13510, Alfa Aesar, Haverhill, MA) and verapamil hydrochloride (VER; Cat. No. V0118, TCI America, Oregon, USA) were prepared as stock solutions in dimethylsulfoxide (DMSO), filtered through 0.22 μm syringe filters and stored as aliquots at −20 °C. Dissolution of piperine at each concentration used in assays was verified by recording the clarity of the culture medium after addition of piperine and stirring or vortexing. A cloudy precipitate formed immediately in the culture medium at concentrations above 200 µg/mL, indicating loss of solubility. Piperine remained completely soluble in culture media at concentrations ≤ 200 µg/mL and no precipitate was observed even after incubation for the duration of the assay. All assays were performed only with piperine at concentrations ≤ 200 µg/mL. Ethidium bromide (EtBr) stock solution was stored in the dark at room temperature and protected from light. Ciprofloxacin (CIP; 98%; Thermo Fisher Scientific), clarithromycin (CLA; > 96%; Alfa Aesar, Haverhill, MA), amikacin (AMI; MP Biomedicals, Solon, OH) and cefoxitin (CEF; TCI America, > 98%) stock solutions were prepared in DMSO, filter-sterilized and stored as aliquots at −20 °C. Dimethyl sulfoxide (DMSO; MP Biomedicals), Tween 80 and Resazurin sodium salt were obtained from Thermo Fisher Scientific.

### 2.3. Assessment of the minimum inhibitory and bactericidal concentrations of antimicrobials

*M. abscessus* cultivated to mid-log phase in Middlebrook 7H9 medium supplemented with Tween 80 and ADC was subsequently diluted 1:100 into CAMHB containing 10% (v/v) ADC to evaluate the minimum inhibitory concentrations (MICs) and minimum bactericidal concentrations (MBCs) of the antimicrobials utilized in this study. All assays were conducted in triplicate using 96-well plates (Thermo Fisher Scientific; Cell Culture-Treated, Flat-Bottom Microplate; Cat. No. FB012931) with each well containing the respective antimicrobial and 100 µL of the culture, approximately 5 x 10^4^ colony-forming units, in a total volume of 200 µL. Wells containing cells without antimicrobials or sterile medium served as controls. The antibiotics CIP, CLA, AMI and CEF were evaluated at concentrations ranging from 0.25X to 40X of their respective MICs (CIP, 0.5 µg/mL; CLA, 1 µg/mL; AMI, 16 µg/mL; CEF, 16 µg/mL), which have been reported for susceptible, rapidly growing, nontuberculous mycobacteria [32]. Following the addition of the antimicrobials, the plates were incubated at 37˚C for 3 days. For MIC determination, 25 µL of sterile 0.02% (w/v) resazurin (final concentration 88 µM) was added into each well, and the plates were incubated again for 24 hours at 37˚C. The resazurin (Alamar blue) assay was utilized for colorimetric assessment of cell viability with viable cells metabolizing the resazurin (blue) to resorufin (pink), whereas non-viable cells remained blue. The lowest concentration at which the wells remained blue was recorded as the MIC for the respective antimicrobial. MICs were determined based on three biological independent experiments, each comprising triplicate samples.

MBCs were assessed by subculturing 20 µL of cultures exposed to antimicrobials for 3 days into 180 µL of fresh CAMHB containing 10% ADC in new 96-well plates, followed by an additional 3-day incubation at 37˚C. The resazurin assay was repeated as described previously, and MBCs were determined accordingly. The lowest concentration that impeded color change from blue was designated as the MBC for each respective antimicrobial. Three independent experiments with samples in triplicate were conducted to establish MBCs.

*M. abscessus* cells in log-phase were diluted 1:100 into Middlebrook 7H9 medium containing Tween 80 and ADC enrichment. Piperine was added at sub-MIC levels (10 µg/mL, 50 µg/mL or 100 µg/mL). Controls received equivalent volume of DMSO. The DMSO concentration was 2% (v/v). Cells were incubated with shaking at 37 °C. Growth was measured using optical density at 600 nm (OD_600_) over 8 days. Experiments were performed independently three times with duplicates in each experiment. Colony-forming units (CFUs) were determined using standard procedures. *M. abscessus* cells were grown to log-phase in Middlebrook 7H9 medium. The cells were then diluted 1:100 in CAMHB with piperine at 100 µg/mL and were incubated for 3 days at 37 °C. Controls were exposed to corresponding volumes of DMSO solvent. The DMSO concentration was 2% (v/v). After 3 days, the cells were serially-diluted and plated on Middlebrook 7H10 agar plates without piperine. CFUs were enumerated after 4 days of incubation at 37 °C. Experiments were performed independently three times with duplicates in each experiment.

### 2.4. Effects of piperine on *M. abscessus* biofilm formation and eradication

*M. abscessus* log-phase cultures were diluted 1:100 in biofilm-stimulating medium containing piperine at the indicated concentrations. Control wells contained cells without piperine. The plates were then placed in plastic bags to reduce evaporation and incubated for 3 days at 37˚C without shaking. Post-incubation, the medium and floating cells were discarded and the wells were gently rinsed with water to clear floating *M. abscessus* cells. Biofilm formation was quantified using the crystal violet assay, as described earlier [9]. The concentration of piperine that inhibited biofilm formation by 50% compared to controls was determined as the minimum biofilm inhibitory concentration (MBIC_50_). The data from three independent biological experiments, each performed in triplicate, is shown.

To assess the minimum biofilm eradication concentration (MBEC) of piperine against *M. abscessus*, the cells were allowed to form biofilms without antimicrobials for 3 days. After biofilm development, floating cells were removed, and piperine was added to evaluate its effects on the dispersal of established biofilms for another 3 days. Following this second incubation, the medium and any floating cells were discarded and the adhered biofilms were rinsed gently three times with distilled water. Biofilm formation was quantified using the crystal violet assay. Three independent biological experiments were performed.

### 2.5. Radioactive fatty acid incorporation into lipids by *M. abscessus*

The metabolic incorporation of radiolabeled palmitic acid by *M. abscessus* cells in log-phase growth or induced to form biofilms for 7 days was examined using a modification of our procedures reported previously [9]. Briefly, *M. abscessus* in log-phase growth was pre-incubated for 5 min with 45 µg/mL piperine at (0.23X MIC) or DMSO prior to incorporation of [1-^14^C]palmitic acid (0.5 µCi/mL cells; 56.67 mCi/mmol; Perkin Elmer Health Sciences Inc., Shelton, CT) for 6 h with shaking at 37 °C. *M. abscessus* was induced to form biofilms in our biofilm-stimulating medium in 24-well tissue-culture plates for 7 days in the presence of 45 µg/mL PIP or equivalent volume of DMSO (control). Then, biofilm cell aggregates were collected by centrifugation at 300 X g, for 10 min and supernatants containing floating cells were discarded. The cell pellets were resuspended in 3.5 mL 7H9 + 0.05% (w/v) Tween 80 without ADC and metabolically radiolabeled with ^14^C-palmitic acid as described above. After radiolabeling, cells were collected by centrifugation at 5000 x g for 10 min. Lipids were extracted and analyzed by silica-thin layer chromatography (TLC) as described previously [9]. Briefly, equal amounts of total lipids (approximately 50,000 dpm per sample) from each sample were analyzed on TLC. Radiolabeled neutral lipids were resolved in a solvent system comprised of hexane:diethyl ether:acetic acid (80:20:2, v/v/v). Radiolabeled polar lipids were resolved on TLC using chloroform:methanol:water (65:25:4, v/v/v) as solvent system. TLC plates were exposed to autoradiographic film. Radioactive bands corresponding to triacylglycerol (TAG; R_f_ ~ 0.4), glycopeptidolipids (GPL; R_f_ ~ 0.78), trehalose dimycolate (TDM; R_f_ ~ 0.71), trehalose monomycolate (TMM; R_f_ ~ 0.53); phosphatidylethanolamine (PE; R_f_ ~ 0.41); phosphatidylglycerol/ cardiolipin (PG/CL; R_f_ ~ 0.33); phosphatidylinositol/ phosphatidylinositol mannosides (PI/PIMs; R_f_ < 0.25) were identified based on their previously-reported R_f_ values in respective TLC solvent systems [9,33,34]. The lipid bands were scraped from the TLC plates and radioactivity was measured by liquid scintillation counting. All radiolabeling experiments were performed independently three times (three biological replicates) with duplicates in each experiment.

### 2.6. Efflux activity assay

The effect of piperine on the efflux activity of *M. abscessus* was investigated using the ethidium bromide (EtBr) efflux assay using a modification of our previously reported procedures [9]. Briefly, *M. abscessus* grown to mid-log phase in Middlebrook 7H9 containing Tween 80 and ADC was collected by centrifugation and resuspended in 7H9 containing 0.05% (w/v) Tween 80 without ADC to obtain an OD_600_ of 0.4. To block initial efflux and allow cells to load up with EtBr, cells were incubated with 150 µg/mL verapamil (0.15X MIC) and 4 µg/mL EtBr at 25 °C for 1 h. The EtBr cells were then harvested by centrifugation and resuspended in fresh 7H9 containing Tween 80 without ADC. The cells were then warmed to 37 °C and added to wells of a black 96-well plate containing piperine at the indicated concentrations or equivalent volume of DMSO as solvent control. Verapamil was used as positive control at 512 µg/mL (0.5X MIC). Intracellular EtBr fluorescence was measured at indicated time-points using an Agilent BioTek Synergy LX fluorescence plate reader (Agilent Technologies, Santa Clara, CA). Fluorescence at 590 nm was measured after excitation at 530 nm. Relative fluorescence values were obtained by normalizing the fluorescence at each time point with the fluorescence at the initial time point, respectively for each sample. Efflux activity at the end-point of the assay was calculated by dividing the fluorescence at 90 min by the fluorescence at 0 min for respective samples. Values obtained were subtracted from 1 and the values for the piperine-treated samples were divided by those of the control samples without piperine and multiplied by 100 to obtain “Efflux Activity (% of control at 90 min).

### 2.7. Assessment of combinatory effects against *M. abscessus* using the checkerboard method

The combinatory effect of piperine with the four antibiotics CIP, CLA, AMI and CEF was assessed using the two-dimensional checkerboard assay. *M. abscessus* cells were cultured to mid-log phase under the conditions outlined previously, then diluted 1:100 into CAMHB supplemented with 10% ADC, and 100 μL was added to each well. Serial dilutions of antimicrobial “A” (PIP) was added along the x-axis of the 96-well plates, while serial dilutions of antimicrobial “B” (CIP, CLA, AMI, or CEF) were added along the y-axis. Control wells lacked either antimicrobial or both. The DMSO concentration in checkerboard assays was ≤ 8% (v/v). Each well contained a total volume of 200 μL. The plates were sealed in plastic bags and incubated at 37˚C for 3 days. MICs were determined using resazurin as described above. The MIC of each antibiotic was assessed across varying concentrations of the antibiotic and piperine. The lowest concentration of an antibiotic alone or in combination with piperine (at ≤ 0.5X of its MIC) that caused a well to retain blue color was taken as the MIC for the antibiotic alone or with piperine, respectively. *M. abscessus* maintained complete viability at concentrations of 0.5X MIC for each antimicrobial tested. Fractional inhibitory concentration index (FICI) values and Modulatory Factors (MF) were calculated and interpreted as described earlier [9]. FICI and Modulatory Factor were determined as follows:

MIC_AB_ = MIC_A_ in the presence of B at ≤ 0.5X MIC_B_
MIC_BA_ = MIC_B_ in the presence of A at ≤ 0.5X MIC_A_
FIC_A_ = MIC_AB_/ MIC_A_
FIC_B_ = MIC_BA_/ MIC_B_
Fractional Inhibitory Concentration Index (FICI) = FIC_A_ + FIC_B_
Modulatory Factor = MIC of antibiotic/ MIC of antibiotic + EPI

As an example, for CIP, FICI and Modulatory Factor were calculated as follows:

MIC_A_ = 200 µg/mL
MIC_B_ = 10 µg/mL
MIC_AB_ = 100 µg/mL
MIC_BA_ = 4 µg/mL
FIC_A_ = MIC_AB_/ MIC_A_ = 100/200 = 0.5
FIC_B_ = MIC_BA_/ MIC_B_ = 4/10 = 0.4
FICI = FIC_A_ + FIC_B_ = 0.5 + 0.4 = 0.9
Modulatory Factor = MIC_B_/ MIC_BA_ = 10/4 = 2.5

### 2.8. Statistical analyses

All experiments in this study were performed independently three times (three biological replicates) with triplicates or duplicates within each experiment, unless specifically stated otherwise. Significance was determined by One-Way ANOVA and post-hoc Tukey test using SigmaPlot 14.5. Experiments involving radioactive lipid analysis were performed independently three times. Data from a representative experiment with values as average ± standard deviation from duplicate samples is shown. Significance determined using Student’s t-Test (two-tailed, unpaired).

## 3. Results

### 3.1. Piperine inhibits *M. abscessus* growth and biofilm formation at sub-MIC concentrations

**Fig 1:**
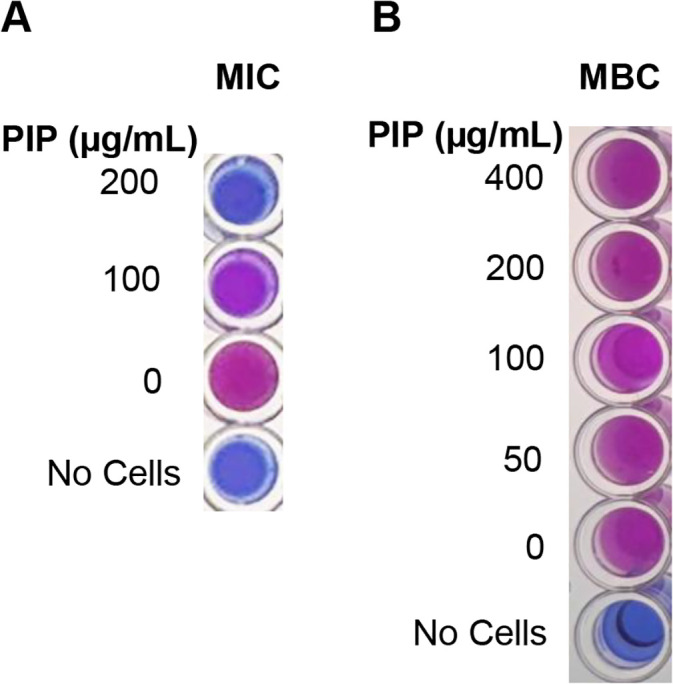
Piperine inhibits cellular metabolism in *M. abscessus.* **A,** Log-phase, cells of *M. abscessus* were exposed to piperine at the indicated concentrations in cation-adjusted Mueller-Hinton broth (CAMHB) for 3 days and then stained with resazurin for 24 h. Pink color signifies active cellular metabolism and blue color signifies a lack of cellular metabolism which indicated the minimum inhibitory concentration (MIC). Three independent experiments were performed. **B,** Minimum bactericidal concentration (MBC) was determined by diluting the cells exposed to piperine for 3 days at the indicated concentrations into CAMHB without piperine and incubated for 3 more days prior to staining with resazurin for 24 h. Three independent experiments were performed.

It has been shown earlier that piperine enhances the efficacy of clarithromycin against *M. abscessus* and inhibits biofilm formation in *M. tuberculosis* [28,31]. However, the effect of piperine on cell viability and biofilm formation in *M. abscessus* has not been investigated. We exposed log-phase *M. abscessus* to piperine for 3 days in CAMHB and determined its MIC (Fig 1A; Table 1). Piperine inhibited cell metabolism at 200 µg/mL, as indicated by the inability of the cells to metabolically convert the resazurin dye to a pink color. When cells incubated with piperine for 3 days were then diluted 10-fold into fresh CAMHB without piperine and incubated for 3 more days, the resazurin assay indicated the presence of living cells capable of active metabolism (Fig 1B; Table 1).

**Table 1:** Effect of piperine on *M. abscessus* viability and biofilm formation.

MIC^a^ (µg/mL)	MBC^b^ (µg/mL)	MBIC_50_^c^ (µg/mL)
200	>400	10

^a^MIC (Minimum Inhibitory Concentration) and ^b^MBC (Minimum Bactericidal Concentration) were determined using log-phase *M. abscessus* in CAMHB. ^c^MBIC_50_ (concentration that inhibited biofilm formation by 50% compared to control) was determined using *M. abscessus* incubated under biofilm-forming conditions.

**Fig 2:**
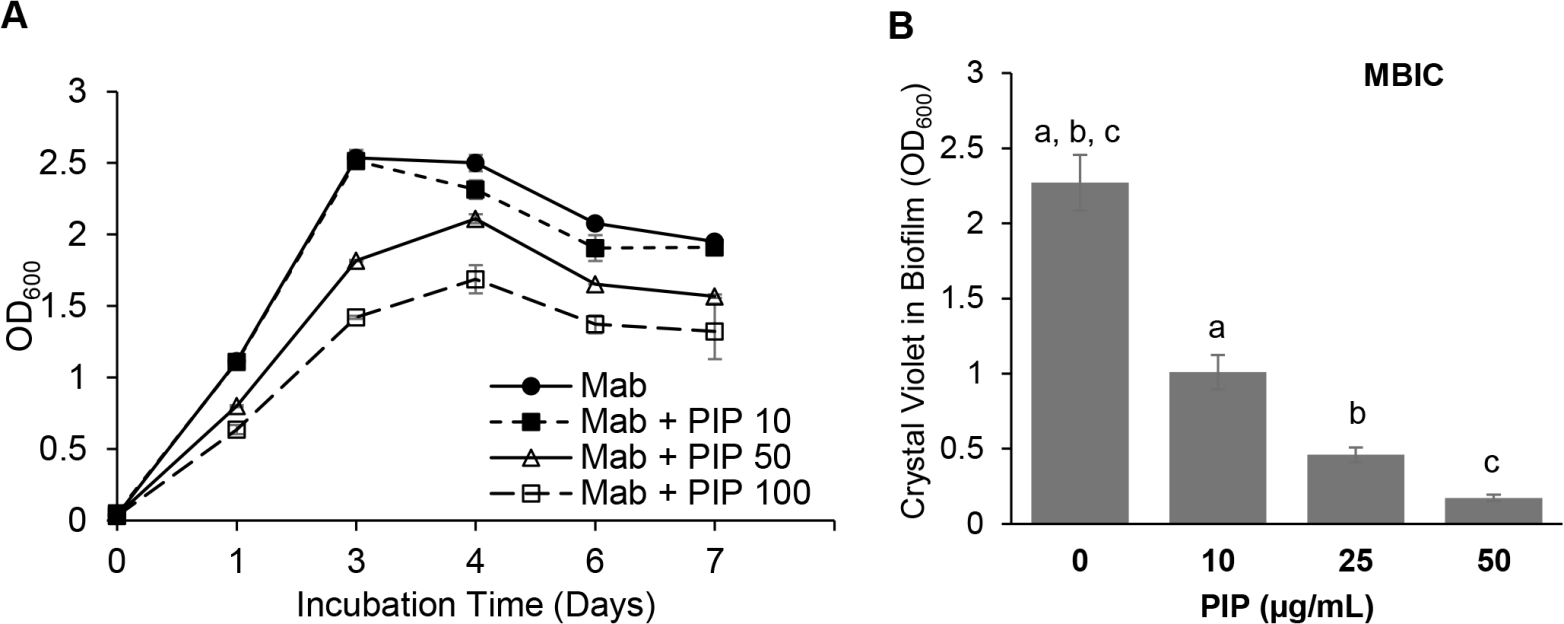
Piperine inhibits growth and biofilm formation by *M. abscessus* at concentrations below MIC. **A.** *M. abscessus* cells in log-phase were incubated with dimethyl sulfoxide (“Mab”) or piperine at sub-MIC levels (100 µg/mL, “PIP 100”; 50 µg/mL, “PIP 50”;10 µg/mL, “PIP 10”) in a shaker at 37 °C. Growth was measured using optical density at 600 nm (OD_600_) over 7 days. Three independent experiments were performed. Data is shown as average ± SD from duplicates in a representative experiment. p < 0.0001: Mab vs. Mab + PIP 100; p < 0.05: Mab vs. Mab + PIP 50 and Not Significant: Mab vs. Mab + PIP 10 from 3 to 7 days. **B,** *M. abscessus* was incubated with piperine under biofilm forming conditions for 3 days. Crystal Violet assay was performed to measure adhered biofilm levels. Three independent experiments were performed. Values from a representative experiment are shown as average ± SD from triplicates. One-Way ANOVA and post-hoc Tukey test performed. a, b, c: p < 0.001 vs. control without piperine.

The effect of piperine on the growth of *M. abscessus* in liquid culture was examined. We observed that sub-MIC levels of piperine were able to significantly inhibit the growth of log-phase cultures of *M. abscessus* in Middlebrook 7H9 broth containing Tween 80. Optical density measurements showed that growth was inhibited by piperine to about 67% of control at 4 days (Fig 2A). We performed agar plating of *M. abscessus* exposed to piperine for 3 days to determine colony-forming units (CFUs). We found that piperine at 100 µg/mL decreased the CFUs on Middlebrook 7H10 agar plates (which did not contain piperine) by one order of magnitude to 5 [± 1] x 10^7^ CFUs/mL, when compared to control cells treated with DMSO solvent (7 [± 0.6] x 10^8^ CFUs/mL). Thus, piperine reduced the living cell population by more than 10-fold but did not eliminate all cells under our assay conditions. At concentrations above 200 µg/mL, piperine did not completely dissolve into CAMHB and showed inferior inhibitory characteristics.

We examined whether piperine could inhibit the formation of biofilms in *M. abscessus* by incubating cells with piperine in biofilm-stimulating medium, that we have previously demonstrated to stimulate biofilm formation by *M. abscessus* [9]. We observed that sub-MIC concentrations of piperine effectively inhibited biofilm formation (Fig 2B). As shown in Table 1, at a concentration of 10 µg/mL, piperine inhibited biofilm formation by 50% compared to untreated controls. In contrast to its ability to inhibit de novo biofilm formation, piperine was not able to eradicate preformed 3-day biofilms at concentrations up to 2X its MIC in our assays.

### 3.2. Piperine dysregulates lipid biosynthesis in biofilm-forming *M. abscessus*

**Fig 3:**
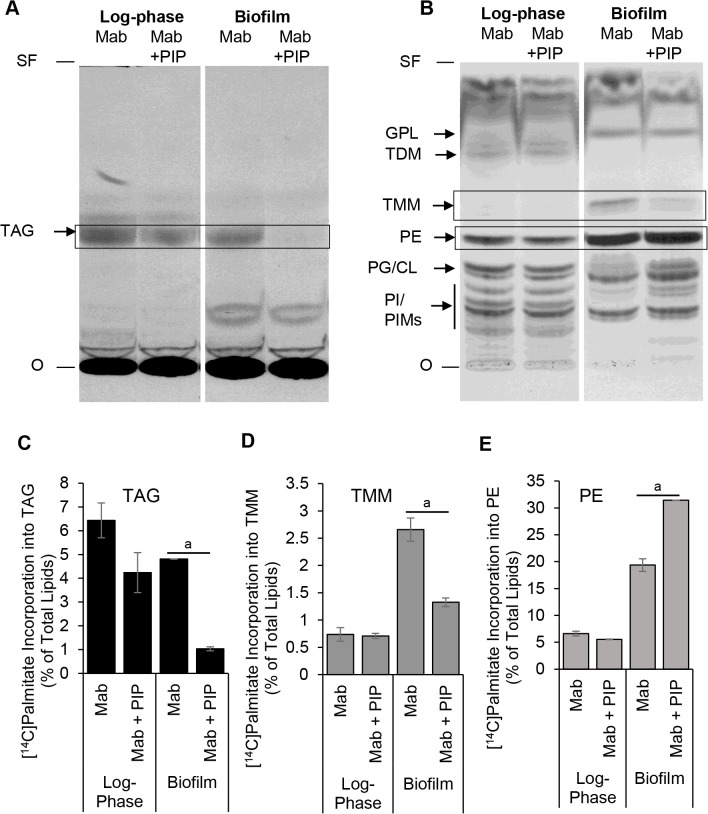
Metabolic incorporation of radiolabeled palmitic acid into triacylglycerol and phospholipids in biofilm-forming *M. abscessus* is dysregulated by piperine. *M. abscessus* cells in log-phase growth stage or after 7-days in biofilm-forming conditions were incubated without or with sub-MIC levels of piperine. The cells were metabolically radiolabeled with ^14^C-palmitic acid for 6 h. Cellular lipids were extracted and equal amounts of radioactivity across samples were analyzed by silica-TLC. Three independent experiments were performed. Autoradiograms of TLC plates from representative experiments shown. **A,** Neutral lipids were resolved in a solvent system comprised of hexane:diethyl ether:acetic acid (80:20:2, v/v/v). TAG, triacylglycerol (R_f_ ~ 0.4). SF, solvent front; O, sample loading zone. **B,** Polar lipids were resolved using chloroform:methanol:water (65:25:4, v/v/v) as solvent system. GPL, glycopeptidolipids (R_f_ ~ 0.78); TDM, trehalose dimycolate (R_f_ ~ 0.71); TMM, trehalose monomycolate (R_f_ ~ 0.53); PE, phosphatidylethanolamine (R_f_ ~ 0.41); PG/CL, phosphatidylglycerol/ cardiolipin (R_f_ ~ 0.33); PI/PIMs, phosphatidylinositol/ phosphatidylinositol mannosides (R_f_ < 0.25). Boxes indicate lipids that showed significant changes due to piperine treatment of cells across three experiments. **C-E,** Normalized quantification of boxed lipids from a representative experiment. Radioactivity in TAG **(C)**, TMM (**D**) and PE (**E**) is shown as a percent of radioactivity in the respective total lipid extract. Average ± SD of duplicates from a representative experiment is shown. Mab, *M. abscessus*; PIP, piperine. Student’s *t*-Tes*t* was performed to determine significance (*M. abscessus* vs. *M. abscessus* + PIP). a: p < 0.05.

It has been shown that *M. abscessus* utilizes exogenous fatty acids for lipid biosynthesis in an environment such as the phagolysosome which is physiologically relevant to its infection process in the human body [35]. We have demonstrated previously that lipid biosynthesis from fatty acid precursor in *M. abscessus* during biofilm formation is affected by the efflux pump inhibitor naphthylmethylpiperazine [9]. The effect of piperine on lipid biosynthesis in *M. abscessus* during biofilm formation has not been reported. Therefore, we investigated whether piperine affects the biosynthesis of neutral and polar lipids in *M. abscessus* from a fatty acid precursor. Radiolabeled ^14^C-palmitic acid was used to metabolically label the lipids of *M. abscessus* in a log-phase state and during biofilm formation in the absence and presence of piperine. We found that piperine severely inhibits the biosynthesis of the neutral storage lipid triacylglycerol (TAG) in biofilm-forming *M. abscessus* (Fig 3A). Piperine inhibited the incorporation of the radiolabeled fatty acid into TAG in biofilm-forming *M. abscessus* by 80% from nearly 5% of total lipids to 1% (Fig 3C). Piperine also dysregulated the biosynthesis of polar lipids from exogenous, radiolabeled fatty acids (Fig 3B). We observed that piperine inhibited the biosynthesis of the polar lipid trehalose monomycolate (TMM) in biofilm-forming *M. abscessus* by 50% from 2.6% to 1.3% of total lipids (Fig 3D). However, piperine stimulated the biosynthesis of the major polar lipid PE in biofilm-forming *M. abscessus* from 20% to more than 30% of total cellular lipids (Fig 3E).

### 3.3. Efflux activity in *M. abscessus* is inhibited by piperine

**Fig 4:**
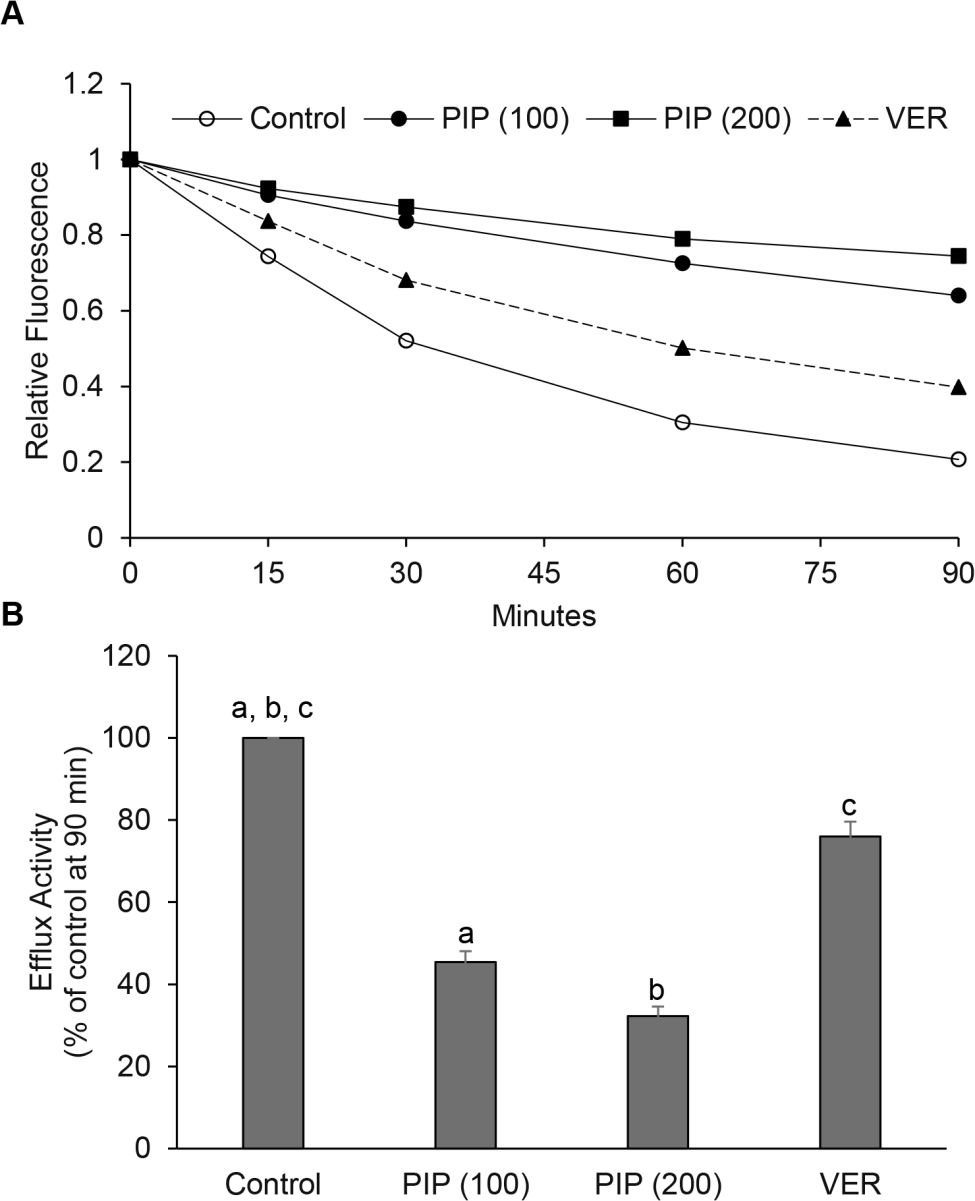
Efflux activity of *M. abscessus* is inhibited by piperine. **A,** Decrease in intracellular fluorescence of *M. abscessus* cells preloaded with EtBr and subsequently incubated with piperine at the indicated concentrations or with dimethyl sulfoxide as control. Verapamil was used as positive control at 512 µg/mL (0.5X MIC). Intracellular fluorescence values at indicated time-points relative to initial time are shown as average of triplicates from a representative experiment. Three independent experiments were performed. **B,** Efflux activity is shown as percent of untreated control at 90 min. One-Way ANOVA and post-hoc Tukey test performed. a,b: p < 0.0001 vs. control; c: p < 0.001 vs. control.

Piperine has been shown to inhibit efflux activity in *M. tuberculosis* and *M. smegmatis* [24,25]. The potential efflux inhibitory activity of piperine in *M. abscessus* has not been reported. We investigated whether the ability of *M. abscessus* to efflux ethidium bromide (EtBr) was inhibited by piperine. *M. abscessus* cells in log-phase growth were examined for efflux activity in the presence of piperine. We observed that piperine at 0.5X MIC inhibited EtBr efflux by 55%. At its MIC, piperine inhibited EtBr efflux by nearly 70% (Fig 4). In comparison, the efflux pump inhibitor verapamil at 0.5X MIC showed only a 20% inhibition of efflux activity, in our assays.

### 3.4. Piperine enhances antibiotic efficacy against *M. abscessus*

Piperine has been reported to improve the efficacy of clarithromycin against *M. abscessus* [31]. However, the effect of piperine on ciprofloxacin, amikacin and cefoxitin, which are commonly used to treat infections by *M. abscessus*, have not been reported. Therefore, the ability of piperine to potentiate the actions of antibiotics against log-phase *M. abscessus* was investigated. We performed two-dimensional checkerboard assays to determine the effects of piperine on the MICs of four antibiotics commonly used against this pathogen. Our data indicated that piperine and the four antibiotics exhibited FICI values of 1 (Table 2). We interpreted this as “No Interaction” based on established criteria in literature [36]. However, piperine was very effective at decreasing the MICs of the antibiotics against *M. abscessus*. The MIC of clarithromycin was decreased by nearly 17-fold and the MICs of amikacin and cefoxitin were decreased by approximately 5-fold (Table 3). The MIC of ciprofloxacin decreased by 2.5-fold in the presence of piperine.

**Table 2:** Combinatory effects of piperine and antibiotics against *Mab.*

Combination (PIP + Antibiotic)	FICI	Combinatory Effect
PIP (100 µg/mL) + Ciprofloxacin (4 µg/mL)	0.9	No interaction
PIP (100 µg/mL) + Clarithromycin (0.3 µg/mL)	1	No interaction
PIP (100 µg/mL) + Amikacin (2.5 µg/mL)	1	No interaction
PIP (100 µg/mL) + Cefoxitin (35 µg/mL)	1	No interaction

Checkerboard assays were performed with the log-phase *M. abscessus* in CAMHB.

**Table 3:** Effect of piperine on the MICs of antibiotics against *Mab.*

	Antibiotic MIC (µg/mL)		
	Piperine 0 µg/mL	Piperine 100 µg/mL	Modulatory Factor
Ciprofloxacin	10	4	2.5
Clarithromycin	10	0.6	16.7
Amikacin	12	2.5	4.8
Cefoxitin	200	35	5.7

Modulatory Factor = MIC of antibiotic alone/ MIC of (antibiotic + piperine). MIC assays were performed with log-phase *M. abscessus* in cation-adjusted Mueller-Hinton broth.

## 4. Discussion

The ability of microorganisms to exist as biofilms as a part of their natural life cycle in the various habitats on earth has been recognized and studied extensively over the past few decades [37]. It is well recognized that microbial biofilms play important roles in human disease. The extracellular polymeric substances produced by the microorganisms in biofilms forms a matrix that serves as a protective layer and acts like a sponge which sequesters extraneous molecules and particles of various kinds [38].

Mycobacteria form biofilms and the obvious importance of this characteristic in mycobacterial pathogenesis has led to several studies on the genetic requirements for biofilm formation [17,39]. *M. tuberculosis* grown under biofilm-inducing conditions was reported to contain TAG and trehalose dimycolates (TDM) [39]. A recent study reported that piperine enhanced the efficacy of clarithromycin against *M. abscessus* [31]. An earlier study showed that piperine inhibited biofilm formation in *M. tuberculosis* [28]. However, the effect of piperine on biofilm formation in *M. abscessus* has not been reported. We postulated that piperine might display inhibitory effects on critical pathogenic mechanisms in *M. abscessus*. The effects of piperine on the biosynthesis of lipids during biofilm formation in mycobacteria or any other bacteria have not been reported. Therefore, we investigated whether piperine affected such lipid biosynthesis from exogenously-supplied fatty acids in *M. abscessus* under the biofilm-forming conditions described in this study. In *M. tuberculosis*, TMM functions as a structural component of the cell wall and also serves as an intermediate molecule in the biosynthesis of TDM which is a major component of the mycomembrane. This pathway is an essential one for *M. tuberculosis* viability and is a target of anti-tuberculosis drugs [40,41]. *M. abscessus* was shown to incorporate exogenous fatty acids into lipids found in the extracellular matrix of its biofilms [8]. We have previously shown that studying the metabolic incorporation of radioactive fatty acids during biofilm formation in *M. abscessus* proved to be valuable in detecting the inhibitory effects of efflux pump inhibitor naphthylmethylpiperazine on lipid biosynthesis [9]. Therefore, we used the metabolic incorporation of exogenously-supplied radioactive C16:0 fatty acids as a highly-sensitive method to investigate lipid metabolism during biofilm formation and the effects of piperine in this study. We observed that the biosynthesis of TMM from ^14^C-palmitic acid increased nearly four-fold in biofilm cells to about 2.6% of total cellular lipids compared to log-phase cells (0.7% of total lipids) suggesting its importance in biofilm formation. In biofilm-forming cells, piperine inhibited TMM biosynthesis by 50% (Fig 3B and 3D). Our observation showing that piperine inhibits the biosynthesis of TMM in *M. abscessus* during biofilm formation is a very interesting finding from this study that could have further implications in our understanding of the biogenesis of the cell envelope associated with biofilm formation in *M. abscessus*. In our study, exposure to piperine did not change the smooth colony morphology of *M. abscessus* on Middlebrook 7H10 agar plates.

We observed that in log-phase cells of *M. abscessus*, phosphatidylethanolamine (PE) comprised only about 5% of total lipids synthesized from exogenously-supplied ^14^C-radiolabeled palmitic acid. Remarkably, in biofilm-forming cells the levels of PE biosynthesis increased to about 20% of total lipids becoming the predominant lipid synthesized. Furthermore, piperine increased the radiolabel accumulation in PE to about 30% of total lipids (Fig 3B and 3E). In the extracellular matrix of *M. abscessus* biofilms grown in a synthetic cystic fibrosis medium lipids constituted nearly 51% of the constituents, in contrast to log-phase cells where lipids constituted only 0.1%. TAG was not found in the lipids of the biofilm matrix [8]. The authors suggest that the DOPC phospholipid in the synthetic cystic fibrosis medium served as the precursor for the lipids found in the *M. abscessus* biofilm matrix. Thus, it can be inferred that the exogenous C18-fatty acid in the phospholipid contributed to the biosynthesis of the GPLs, free mycolic acids and PIMs in the extracellular matrix of *M. abscessus* in that study. Under our growth conditions, we observed that the biosynthesis of TAG from exogenously-supplied radiolabeled palmitic acid comprised about 4–6% of total lipids in log-phase and biofilm cells of *M. abscessus* and this was inhibited to about 1% in the presence of piperine at a concentration that inhibited biofilm formation (Figs 2B and 3C). TAG is a storage lipid that has been shown to be accumulated in *M. tuberculosis* during dormancy-inducing conditions and in *M. abscessus* by us and others earlier [34,35]. We had previously shown that TAG biosynthesis in *M. abscessus* was upregulated by naphthylmethylpiperazine [9]. In this study, we show that piperine inhibits the biosynthesis of TAG in *M. abscessus* during biofilm formation. This study is the first to report on the effects of piperine on lipid biosynthesis associated with log-phase cells and with biofilm formation in *M. abscessus*.

Efflux pumps are thought to function not only as drug exporters but also have potential roles in the transport of molecules involved in biofilm formation [15]. The upregulation of bacterial efflux pump gene expression in biofilms and the ability of efflux pump inhibitors to disrupt biofilm formation have been reported [42,43]. Efflux pump inhibitors are known to be effective in tackling antimicrobial drug resistance in *M. tuberculosis* [18]. The efflux pump inhibitors piperine and naphthylmethylpiperazine were shown to inhibit biofilm formation in *M. tuberculosis* [28]. We had reported earlier that naphthylmethylpiperazine inhibited biofilm formation in *M. abscessus* [9]. In a different study, we had reported that fullertubes and fullerenes, which are carbon nanoparticles, inhibited pellicle biofilm formation in *M. smegmatis* [44].

Piperine, an alkaloid derived from black pepper, has been shown to inhibit efflux pump activity and biofilm formation in mycobacteria and other bacteria [23]. Piperine has been shown to increase the production of cytokines and promote proliferation of lymphocytes in mice infected with *M. tuberculosis*. In the same study, the authors showed that piperine significantly improved the therapeutic effect of rifampicin although piperine alone did not decrease the CFUs of *M. tuberculosis* in mice [29]. In *M. tuberculosis*, piperine was reported to inhibit efflux activity and biofilm formation and increase the efficacy of anti-tuberculosis drugs [25,26,28]. Piperine has been reported to inhibit ethidium bromide efflux and increase the efficacy of clarithromycin in nontuberculous mycobacteria [24,27,31]. However, to our knowledge, there are no reports on the effects of piperine on the efflux activity, lipid biosynthesis and biofilm formation in *M. abscessus*. Therefore, in our current study, we examined whether piperine affected these characteristics of *M. abscessus*. Since the effect of piperine on efflux activity in *M. abscessus* has not been reported, we investigated it using the ethidium bromide efflux assay in this study. Our findings which showed that piperine inhibited efflux activity in *M. abscessus* by nearly 70% when used at its MIC demonstrates the efflux inhibitory activity of piperine in this pathogen for the first time (Fig 4). Alternatively, the observed efflux inhibitory property of piperine could be due to the altered properties of the mycobacterial cell envelope caused by the changes in the lipid profile (Fig 3) rather than direct efflux pump effects. This remains to be investigated.

We report here for the first time that piperine at 0.25X MIC inhibits biofilm formation in *M. abscessus* by more than 90% (Table 1; Figs 1A and 2B). The above observations suggest that piperine might be inhibiting the efflux of molecules associated with biofilm formation. Piperine might be inhibiting the transport of molecules synthesized within the cell and transported to the outer mycobacterial surface and secreted into the extracellular matrix of the biofilms. Since piperine inhibited biosynthesis of the neutral lipid TAG and the polar lipid TMM in biofilm-forming cells of *M. abscessus* in this study (Fig 3), it is possible that these lipids are involved in biofilm formation processes. Further studies are needed to investigate these initial findings.

The MICs for piperine against *M. abscessus* have not been reported earlier. In our current study, we found that piperine exhibited an “MIC” of 200 µg/mL. However, piperine did not exhibit bactericidal activity at concentrations up to its solubility limit in the culture medium used in the assay (Fig 1B and Table 1). These observations suggest that piperine blocks vital cellular metabolism leading to a lack of color change of the redox indicator dye resazurin in our assays but does not kill the cells at its MIC. The small fraction of cells that are tolerant of piperine under our assay conditions were able to regrow when diluted in the MBC assay and display their ability to metabolize the resazurin dye (Table 1, Fig 1A, 1B).

Our investigation of the effects of piperine on the growth and viability of log-phase cultures of *M. abscessus* showed that although optical density was decreased by piperine at 100 µg/mL to about 67% of control, CFUs on agar plates decreased by an order of magnitude. CFU measurements are considered the primary metric of cell viability in mycobacteria. Optical density is not ideal for quantitative measurements of cell viability since mycobacteria are prone to cell clumping and dead cell debris could contribute to non-linear measurements of turbidity that do not correspond to viable cell counts on agar plates. The apparent discrepancy between our optical density and CFU measurements could be caused by such factors. At 100 µg/mL, piperine decreased CFUs on agar plates by nearly an order of magnitude but did not kill all the cells. This suggests that a small fraction of the *M. abscessus* population contains cells that are tolerant of piperine at this concentration.

Piperine was recently shown to increase the efficacy of clarithromycin in *M. abscessus* [31]. Our two-dimensional checkerboard assays indicated that piperine showed “No Interaction” with the four antibiotics tested (Table 2). However, piperine decreased the MIC of clarithromycin against log-phase *M. abscessus* by nearly 17-fold (Table 3). This finding is in agreement with the recent report where the authors reported a similar modulatory activity of piperine against clarithromycin against *M. abscessus* [31]. We found that piperine decreased the MICs of amikacin and cefoxitin by approximately 5-fold and that of ciprofloxacin by more than 2-fold (Table 3). This is the first report of the modulatory effects of piperine on the efficacies of ciprofloxacin, amikacin and cefoxitin which are commonly used to treat infections by *M. abscessus*.

The molecular target of piperine in *M. smegmatis* was identified as the GTPase FtsZ [45]. However, it is likely that piperine exhibits the effects observed in this study by its actions on other uncharacterized targets. Further studies are needed to understand such mechanisms of action.

## Supporting information

S1 Fig Supporting Information Fig 1A Checkerboard assay for determining minimum inhibitory concentration. *M. abscessus* in mid-log phase was diluted 1:100 into CAMHB containing 10% (v/v) ADC to evaluate the minimum inhibitory concentrations. The plate was incubated at 37˚C for 3 days. Resazurin was added and the plate was incubated again for 24 hours at 37˚C. The lowest concentration at which a well remained blue was recorded as the MIC for the respective antimicrobial.
(JPG)

S2 Fig Supporting Information Fig 1B Checkerboard assay for determining minimum bactericidal concentration. *M. abscessus* in mid-log phase was diluted 1:100 into CAMHB containing 10% (v/v) ADC to evaluate the minimum inhibitory concentrations. The plate was incubated at 37˚C for 3 days. Cultures exposed to antimicrobials for 3 days were diluted 1:10 into fresh CAMHB containing 10% ADC in new 96-well plates, followed by an additional 3-day incubation at 37˚C. Resazurin was added and the plate was incubated again for 24 hours at 37˚C. Pink color indicates metabolically viable cells.
(JPEG)

S3 Fig Supporting Information Fig 2A Measurement of growth of *M. abscessus* by optical density. *M. abscessus* cells in log-phase were incubated with dimethyl sulfoxide (“Mab”) or piperine at sub-MIC levels (100 µg/mL, “PIP 100”; 50 µg/mL, “PIP 50”;10 µg/mL, “PIP 10”) in a shaker at 37 °C. Growth was measured using optical density at 600 nm (OD_600_) over 7 days. Three independent experiments were performed. Data is shown as average ± SD from duplicates in a representative experiment.
(XLSX)

S4 Fig Supporting Information Fig 2B Effect of piperine on biofilm formation by *M. abscessus.* *M. abscessus* was incubated with piperine under biofilm forming conditions for 3 days. Crystal Violet assay was performed to measure adhered biofilm levels. Three independent experiments were performed. Values from a representative experiment are shown as average ± SD from triplicates.
(XLSX)

S5 Fig Supporting Information Fig 3 Piperine inhibits metabolic incorporation of radiolabeled fatty acids into triacylglycerol by *M. abscessus* biofilms. Neutral lipids were resolved in a solvent system comprised of hexane:diethyl ether:acetic acid (80:20:2, v/v/v). TAG, triacylglycerol (R_f_ ~ 0.4). SF, solvent front; O, sample loading zone. Polar lipids were resolved using chloroform:methanol:water (65:25:4, v/v/v) as solvent system. GPL, glycopeptidolipids (R_f_ ~ 0.78); TDM, trehalose dimycolate (R_f_ ~ 0.71); TMM, trehalose monomycolate (R_f_ ~ 0.53); PE, phosphatidylethanolamine (R_f_ ~ 0.41); PG/CL, phosphatidylglycerol/ cardiolipin (R_f_ ~ 0.33); PI/PIMs, phosphatidylinositol/ phosphatidylinositol mannosides (R_f_ < 0.25). Autoradiograms of TLC plates from representative experiments with duplicates are shown.
(PDF)

S6 Fig Supporting Information Fig 4 Piperine inhibits efflux activity in *M. abscessus.* Decrease in intracellular fluorescence of *M. abscessus* cells preloaded with EtBr and subsequently incubated with piperine at the indicated concentrations or with dimethyl sulfoxide as control. Verapamil was used as positive control. Intracellular fluorescence values at indicated time-points relative to initial time are shown as average of triplicates from a representative experiment. Three independent experiments were performed. Efflux activity is shown as percent of untreated control at 90 min.
(XLSX)

S1 Table Supporting Information Fig 3C Quantification of radioactivity incorporated metabolically into TAG. Radioactivity in TAG is shown as a percent of radioactivity in the respective total lipid extract. Average ± SD of duplicates from a representative experiment is shown. Mab, *M. abscessus*; PIP, piperine.
(XLSX)

S2 Table Supporting Information Fig 3D Quantification of radioactivity incorporated metabolically into PE and TMM. Radioactivity in TMM and PE is shown as a percent of radioactivity in the respective total lipid extract. Average ± SD of duplicates from a representative experiment is shown. Mab, *M. abscessus*; PIP, piperine.
(XLSX)
