# Supplementary figures and images for: Piperine inhibits biofilm formation and efflux activity and dysregulates lipid metabolism in Mycobacterium abscessus
Source: PLoS One. 2026 Jan 22;21(1):e0341420. doi: 10.1371/journal.pone.0341420 (PMC12826518; doi:10.1371/journal.pone.0341420)

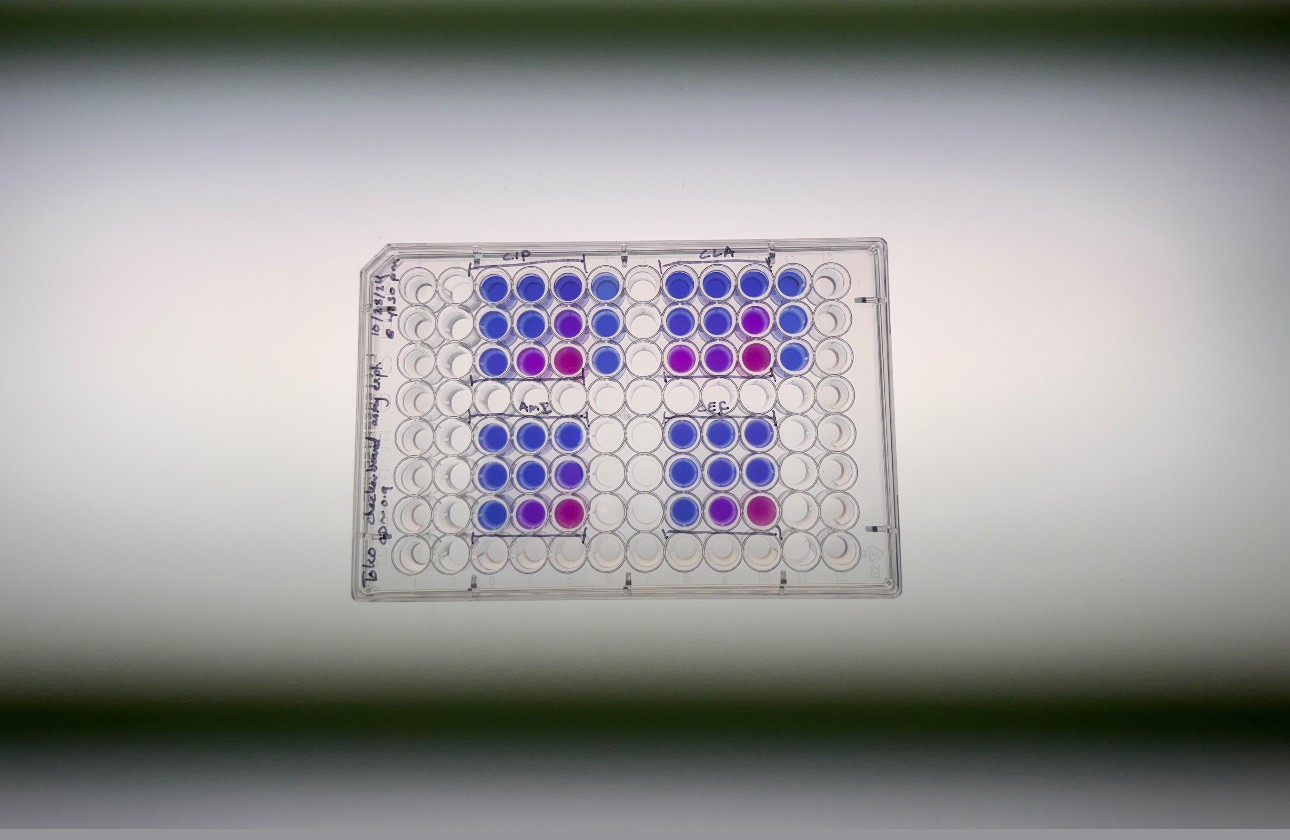

Supplement: S1 Fig — M. abscessus in mid-log phase was diluted 1:100 into CAMHB containing 10% (v/v) ADC to evaluate the minimum inhibitory concentrations. The plate was incubated at 37˚C for 3 days. Resazurin was added and the plate was incubated again for 24 hours at 37˚C. The lowest concentration at which a well remained blue was recorded as the MIC for the respective antimicrobial. (JPG) [file pone.0341420.s001.JPG]

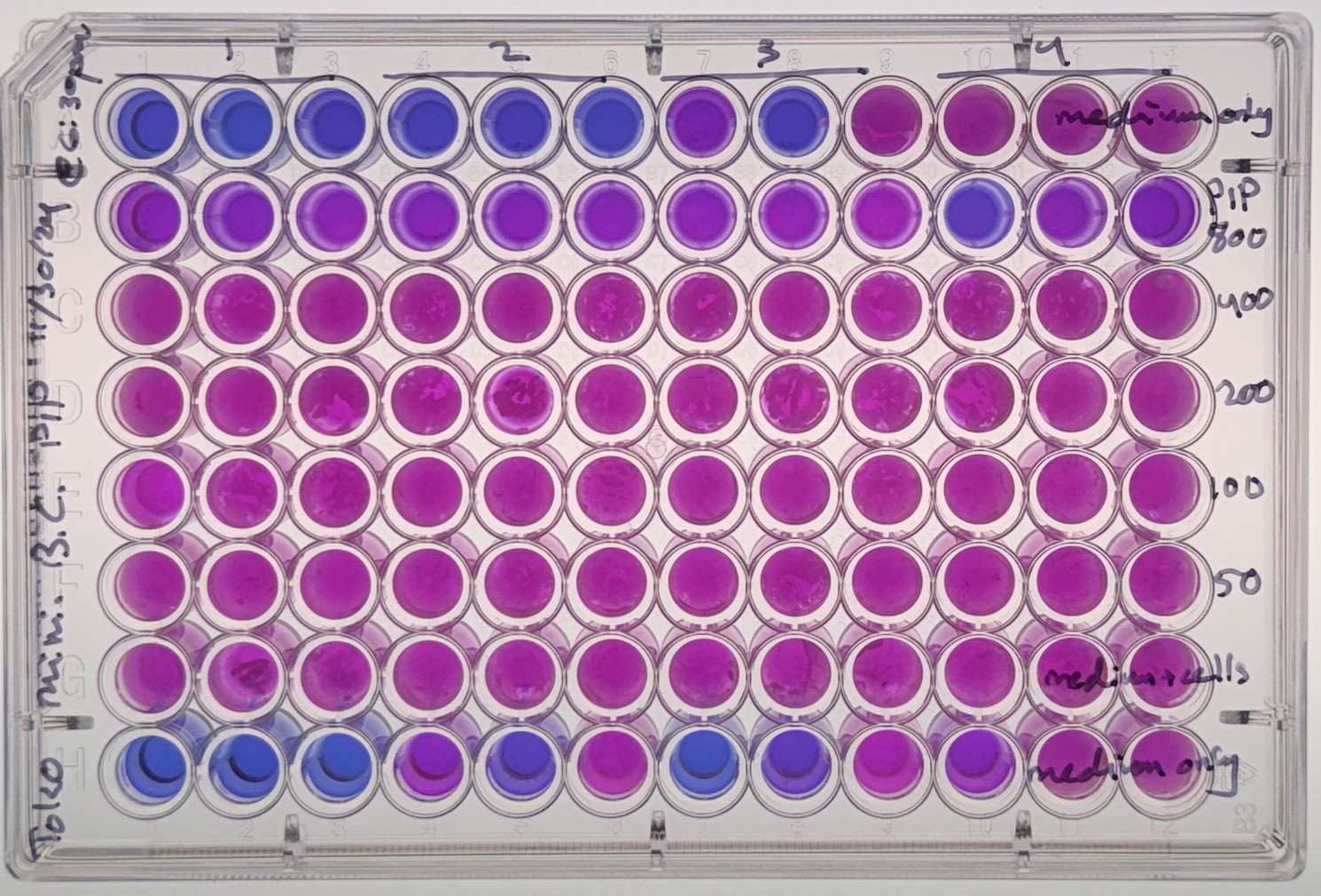

Supplement: S2 Fig — M. abscessus in mid-log phase was diluted 1:100 into CAMHB containing 10% (v/v) ADC to evaluate the minimum inhibitory concentrations. The plate was incubated at 37˚C for 3 days. Cultures exposed to antimicrobials for 3 days were diluted 1:10 into fresh CAMHB containing 10% ADC in new 96-well plates, followed by an additional 3-day incubation at 37˚C. Resazurin was added and the plate was incubated again for 24 hours at 37˚C. Pink color indicates metabolically viable cells. (JPEG) [file pone.0341420.s002.jpeg]

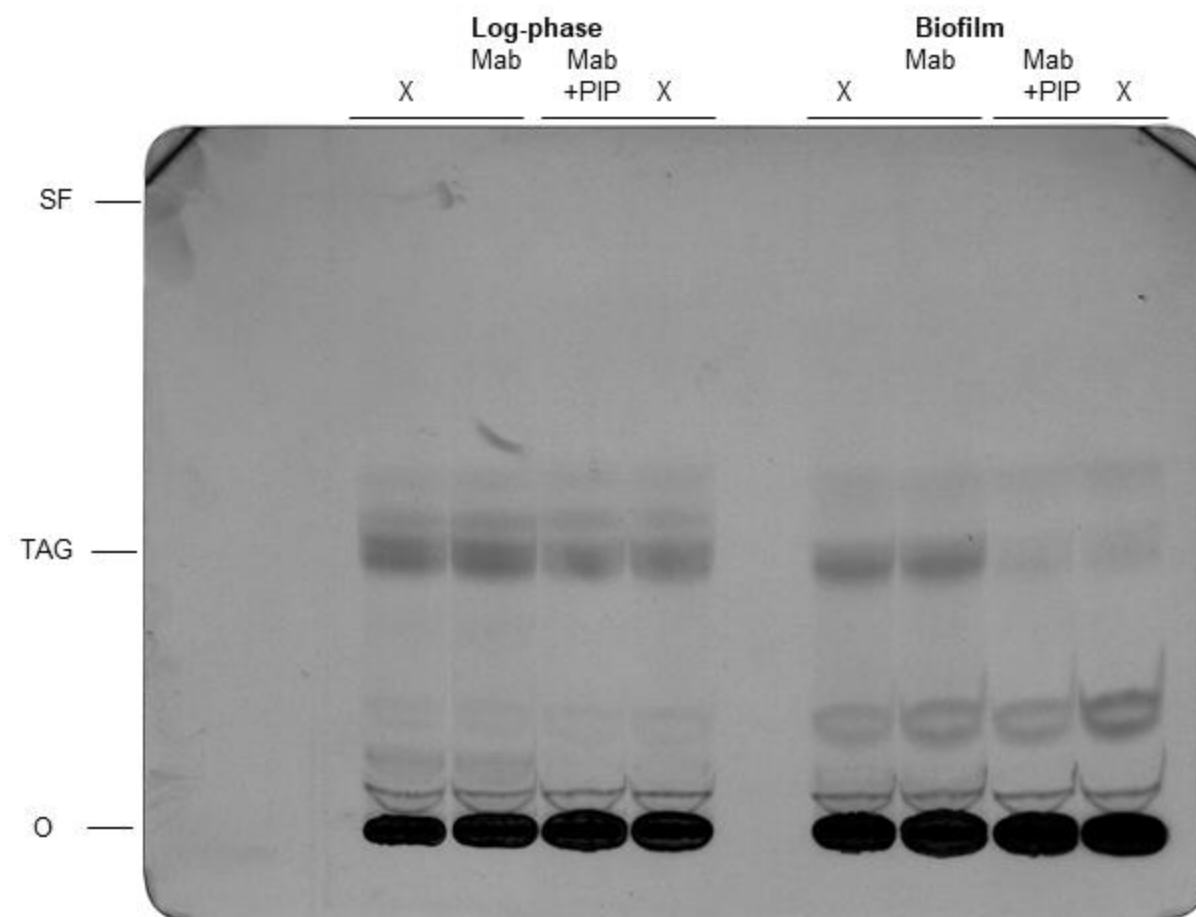

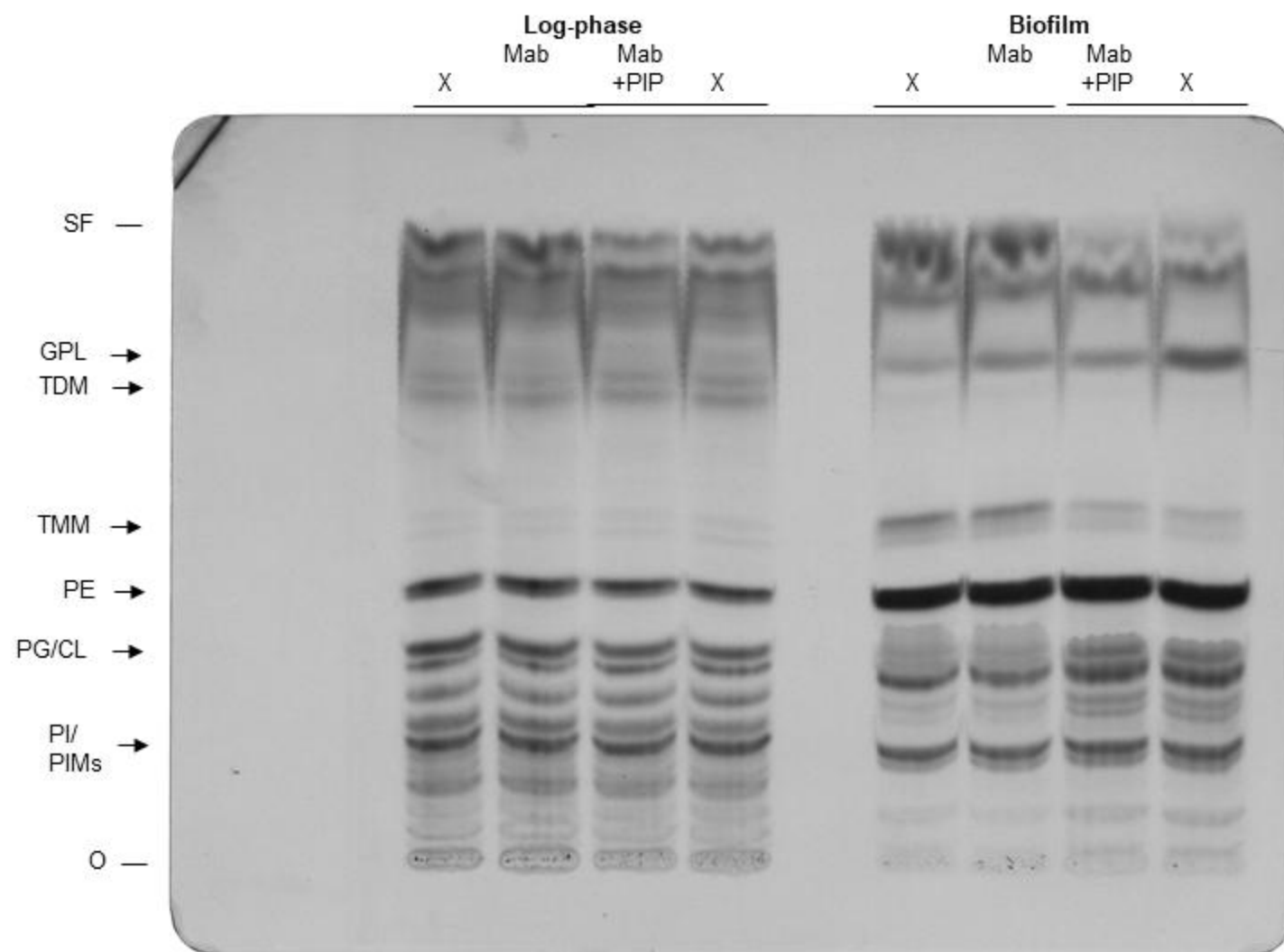

Supplement: S5 Fig — Neutral lipids were resolved in a solvent system comprised of hexane:diethyl ether:acetic acid (80:20:2, v/v/v). TAG, triacylglycerol (Rf ~ 0.4). SF, solvent front; O, sample loading zone. Polar lipids were resolved using chloroform:methanol:water (65:25:4, v/v/v) as solvent system. GPL, glycopeptidolipids (Rf ~ 0.78); TDM, trehalose dimycolate (Rf ~ 0.71); TMM, trehalose monomycolate (Rf ~ 0.53); PE, phosphatidylethanolamine (Rf ~ 0.41); PG/CL, phosphatidylglycerol/ cardiolipin (Rf ~ 0.33); PI/PIMs, phosphatidylinositol/ phosphatidylinositol mannosides (Rf < 0.25). Autoradiograms of TLC plates from representative experiments with duplicates are shown. (PDF) [file pone.0341420.s005.pdf]
